# Comparing 16S rDNA amplicon sequencing and hybridization capture for pea aphid microbiota diversity analysis

**DOI:** 10.1186/s13104-018-3559-3

**Published:** 2018-07-11

**Authors:** Marie Cariou, Céline Ribière, Stéphanie Morlière, Jean-Pierre Gauthier, Jean-Christophe Simon, Pierre Peyret, Sylvain Charlat

**Affiliations:** 1 Laboratoire de Biométrie et Biologie Evolutive, CNRS, UMR 5558, Université de Lyon, Université Lyon 1, 43 Boulevard du 11 novembre 1918, 69622 Villeurbanne, France; 2 Present Address: Department of Biology, University of Namur, Rue de Bruxelles 61, 5000 Namur, Belgium; 3 INRA, MEDIS, Université Clermont Auvergne, 63000 Clermont-Ferrand, France; 4 INRA, UMR 1349 (IGEPP “Institut de Génétique, Environnement et Protection des Plantes”) INRA/Agrocampus Ouest/Université Rennes 1, 35653 Le Rheu, France

**Keywords:** Hybridization capture, Amplicon sequencing, 16S, Pea aphid, Microbiota

## Abstract

**Objective:**

Targeted sequencing of 16S rDNA amplicons is routinely used for microbial community profiling but this method suffers several limitations such as bias affinity of universal primers and short read size. Gene capture by hybridization represents a promising alternative. Here we used a metagenomic extract from the pea aphid *Acyrthosiphon pisum* to compare the performances of two widely used PCR primer pairs with DNA capture, based on solution hybrid selection.

**Results:**

All methods produced an exhaustive description of the 8 bacterial taxa known to be present in this sample. In addition, the methods yielded similar quantitative results, with the number of reads strongly correlating with quantitative PCR controls. Both methods can thus be considered as qualitatively and quantitatively robust on such a sample with low microbial complexity.

**Electronic supplementary material:**

The online version of this article (10.1186/s13104-018-3559-3) contains supplementary material, which is available to authorized users.

## Introduction

High throughput sequencing has revealed that the microbial world is more diverse, more abundant, and more ubiquitous than most had ever imagined [1, 2]. Yet, accurately describing this diversity, meaning to identify which taxa are present in a given environmental extract, and to assess their abundance, remains technically and economically challenging. Metagenomics represents a powerful approach, providing not only a mostly unbiased picture of the taxonomic diversity, but also potentially rich information on the entire gene content. But this approach remains too costly to be applied to many samples in multiplexed sequencing reactions and the bioinformatic treatment is still not trivial. Metabarcoding approaches, that target a taxonomically relevant marker such as the 16S rRNA gene, represent a potential alternative [3]. Amplicon sequencing, described as the high throughput sequencing of tagged PCR amplicons, is a widely used technique that has provided abundant and interesting data, but potentially suffers from the biased affinity of “universal” PCR primers for particular taxonomic groups [3]. Indeed, no genomic region, even in such a constrained locus, is universally conserved, and even slight variation in the primer target regions can introduce biases in the sequencing results, due to the exponential PCR process. In addition, amplicon sequencing shows limits in precise affiliation due to short genomic region explored.

Here we compare amplicon sequencing with DNA capture based on solution hybrid selection (SHS), corresponding to the selective retention of genomic regions matching a set of probes [4]. This method relies on the synthesis of RNA probes complementary to a gene of interest, in this study the 16S rRNA gene. Several properties of DNA capture make it a promising candidate approach for a faithful description of microbial diversity [5, 6]. First, it is possible to introduce in the capture mix a virtually unlimited number of probes to target the gene of interest. Second, this method does not rely on PCR amplification, thus maximising the ability to capture all taxa without bias. Third, it is possible, in particular for the very well-known 16S rRNA gene, to design probes not only based on the known diversity, but also “exploratory probes”, that would hybridise to 16S rDNA sequences that have not been described but might exist, since they would maintain the ribosomal RNA structure.

Following earlier works that have demonstrated the efficiency of DNA capture [6, 7] we compare this method to the now classic amplicon sequencing approach. We use two broadly used combinations of PCR primers and the DNA capture on a control metagenomic sample from the pea aphid *Acyrthosiphon pisum*, of which the microbial composition has been previously described [8]. We first compare the methods qualitatively, to confirm that the 8 bacterial taxa present in the aphid sample are detected, although they were not specifically targeted. We further ask which method is most reliable quantitatively, i.e. provides the most accurate picture of the relative abundance of the most common taxa. We found that the amplicon sequencing and DNA capture approaches produced a similar picture of the microbial diversity and abundance, both qualitatively and quantitatively. Thus, while previous studies have indicated that DNA capture is more effective on complex metagenomic samples [6], it appears that on a sample of low complexity the two techniques are validated, and can be considered as equivalently efficient.

## Main text

### Materials and methods

#### DNA source, 16S rDNA amplification and sequencing

**Fig. 1:**
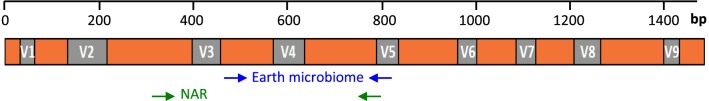
Positioning of the two pairs of primers used for 16S amplification. In blue: earth microbiome primers [9], producing a 300 bp amplicon). In green, NAR primers [10], producing a 450 bp amplicon

The aphid DNA was previously obtained and characterized by Gauthier et al. [8]. DNA was extracted from each individual (whole body) using the QIAGEN DNeasy kit following the manufacturer’s protocol. DNA extractions were then quantified using a Nanodrop spectrophotometer and DNA from 20 individuals (collected in a single field of pea *Pisum sativum*) were normalized and combined to create a DNA pool (App1), that was used in the present study. Two PCR primer pairs for next-generation sequencing-based diversity studies were used (Fig. 1): the earth microbiome primer pair (EM) [9] producing a 292 bp amplicon targeting the hypervariable V4 region (515F: GTGCCAGCMGCCGCGGTAA, 806R: GGACTACHVGGGTWTCTAAT); and the NAR primer pair selected by Klindworth et al. [10] producing a 464 bp amplicon targeting the hypervariable V3 and V4 regions (S-D-Bact-0341-b-S-17: CCTACGGGNGGCWGCAG, S-D-Bact-0785-a-A-21: GACTACHVGGGTATCTAATCC).

PCR was performed in a 25 µl reaction volume, using 0.6 units of Taq DNA polymerase (Expand High Fidelity PCR System, Roche, ref: 11732650001) and 1 µl of DNA template, in the following conditions: 1.5 mM MgCl_2_, 0.125 mM of each dNTP, 0.4 µM reverse and forward primers. The annealing temperature for EM and NAR primer pairs is 50 and 58 °C, respectively. Thermal cycles were as follows: 94 °C for 2 min (94 °C for 30 s, 30 s at the annealing temperature, 72 °C for 1 min) 33 times and 72 °C for 6 min. Each PCR was replicated three times prior to sequencing. Amplicons were processed and deep sequenced on the GS FLX Titanium system at the Environmental Genomics platform of BioGenouest (University of Rennes 1, France, https://geh.univ-rennes1.fr/) as detailed in [8].

#### SHS capture and sequencing

Capture probes were designed using KASpOD [11] and PhylArray [12] and presented in [6]. Adaptor sequences were added to the 5′ and 3′ ends of the oligonucleotides for RNA probes production. These hybrid oligonucleotides consist of 5′-ATCGCACCAGCGTGT(X)CACTGCGGCTCCTCA-3′, with X indicating the specific capture probe sequence. The RNA probes were prepared as described in Ribière et al. [13]. Briefly, in the first step, oligonucleotides were amplified by PCR using primers complementary to the 5′ and 3′ adaptors, to allow double-strand DNA formation. In the second step, agarose gel-purified double-stranded DNA probes were used as template to produce biotinylated RNA probes by in vitro transcription using the MEGAScript^®^T7 kit (Ambion, Life Technologies) and biotin-dUTP (TeBu Bio). Biotinylated RNA probe mix was purified using RNeasy Plus Mini Kit (Qiagen, Hilden, Germany). The concentration and the quality were evaluated with Nanodrop spectrophotometer (ThermoScientific, Wilmington, DE, USA) and on an Agilent Bioanalyzer RNA 6000 Nano chip (Santa Clara, Ca, USA).

16S rRNA gene capture was carried out as described by Denonfoux et al. [7]. Libraries were prepared using Roche GS FLX Rapid Library Preparation Kit (Roche Applied Science) according to the manufacturer’s instructions. First, 500 ng of extracted DNA was sheared by nebulization. DNA fragments were size selected with AMPure beads (Beckman Coulter Genomics). After purification, fragment end repair, and adaptors ligation, the libraries were PCR amplified with the GC-RICH PCR System Kit (Roche Applied Science) using the following primers (TS-PCR Oligo 1 5′-AATGATACGGCGACCAC CGAGA-3′ and TS-PCR Oligo 2 5′-CAAGCAGAAGACGGCA TACGAG-3′) as in [13]. The cycle conditions were 4 min at 94 °C followed by 20 cycles of 30 s at 94 °C, 1 min at 58 °C and 1 min 30 s at 68 °C and a final elongation step at 68 °C for 3 min. The amplified libraries were purified with AMPure beads. 500 ng of amplified library was hybridized to the equimolar mix of biotinylated RNA probes (500 ng) for 24 h at 65 °C. Probe/DNA hybrids were trapped by streptavidin-coated paramagnetic beads (Dynabeads^®^ M-280 Streptavidin, Invitrogen). After two different washing steps (1× SSC, 0.1% SDS for 15 min at room temperature and 0.1× SSC, 0.1%. SDS for 10 min at 65 °C) to remove unbound DNA fragments, the captured DNAs were eluted from the beads using 50 µl of 0.1 M NaOH at room temperature, neutralized with 70 µl of 1 M Tris–HCl, pH 7.5, and purified using the Qiaquick PCR purification kit (Qiagen). Captured DNAs were PCR amplified with the GC-RICH PCR System Kit (Roche Applied Science) following the cycle conditions described for the DNA library amplification but with 25 cycles instead of 20. Ten independent amplifications were conducted on the sample. PCR products were purified on QIAquick columns (Qiagen), size selected on AMPure beads, and pooled. A second round of hybridization and PCR amplification was performed using the amplified gDNA sample obtained after the first hybridization capture. Purified products were pooled together and quantified by spectrophotometry (NanoDrop™ 2000, Thermo Scientific). The DNA quality and size distribution of captured DNA were assessed on an Agilent 2100 Bioanalyzer DNA 12000 chip (Agilent Technologies) and then sequenced using the GS FLX Titanium system on the Macrogen platform (http://www.macrogen.com/).

#### Quantitative PCR

Quantitative PCR was used to produce an unbiased measure of the quantity of the various microbial taxa in our sample, following a previously established protocol [14]. Twenty microliters qPCR reactions were carried out on a LightCycler^®^ 480 II (Roche Diagnostic, Switzerland) with LightCycler 480 SYBR Green I Master (Roche Diagnostic, Switzerland) and specific primers as indicated in Additional file 1: Table S1.

Each reaction consisted of 10 μl of LightCycler 480 SYBR Green I Master (Roche Diagnostic, Switzerland), 0.8 µl of each primer (0.4 µM), 6.4 µl of water and 2 µl of DNA. All samples were analysed in duplicate. For each symbiont, one non-template control (NTC) was used to check the PCR performance. The following LightCycler experiment was used: denaturation program (95 °C for 5 min), followed by 45 cycles of (94 °C for 10 s, 60 °C for 30 s, 72 °C for 20 s with a single fluorescence measurement), and finally a cooling step to 40 °C. Melting curve analysis was conducted to confirm the purity of the qPCR products for each targeted symbiont, at a constant temperature increase (60–95 °C), recording fluorescence every 10 s. To determine the crossing point (CP) for each sample (that is, the point at which the fluorescence rises appreciably above the background fluorescence), a fit point method was applied using the LightCycler software 1.5 (Roche Diagnostic). Data analyses were performed using LightCycler 480 software (Roche Diagnostic, Switzerland). Relative quantity of bacterial gene copy per aphid gene copy was calculated using mathematical delta-delta method [15]. The mean of the two technical replicates per symbiont was used to measure the relative quantity.

### Results and discussion

In the present study, we compared the performance, with respect to microbial community profiling, of two commonly used broad range 16S rDNA primer pairs with 16S rDNA gene capture approach. Table 1 provides a summary of the number of reads per experiment. We first evaluated the suitability of the two strategies to qualitatively describe the composition of this low-complexity insect microbiota. After quality assessment, amplicons and targeted capture reads were aligned to SILVA SSU reference alignment (see Additional file 2 for further details on sequence data processing). In order to accurately compare the different methods, the first step was to retrieve reads overlapping with the hypervariable V4 region of the 16S rDNA gene for defining Operational Taxonomic Units (OTUs) at a 97% sequence identity threshold. Based on this dataset, 8 OTUs were identified using the amplicon data. These OTUs all correspond to known aphid bacterial symbionts [8, 16], respectively *Buchnera aphidicola,* the aphid primary symbiont, and *Hamiltonella defensa*, *Rickettsiella viridis*, *Rickettsia* sp., *Regiella insecticola*, *Fukatsuia* (also known as PAXS or X-type-), *Serratia symbiotica* and *Spiroplasma* sp. One additional lineage from the *Myxococcales* order was detected using the gene capture approach. However, its presence could not be confirmed by targeted PCR, despite repeated attempts based on two different sets of primers (not shown), suggesting these data either stem from contamination or from the extreme sensitivity of gene capture by hybridization. Overall, the amplicon sequencing and gene capture approaches are qualitatively equivalent, producing similar pictures of the microbial diversity present in this sample.

**Table 1:** Number of reads from the different approaches

Method	Raw reads	Reads after QC	Reads after chimera removal	Proportion of rRNA reads (%)
EM primers [9]	3086	3010	3000	100.00
NAR primers [10]	39,551	37,417	37,362	100.00
SHS capture	18,113	12,930	12,930	68.61

**Fig. 2:**
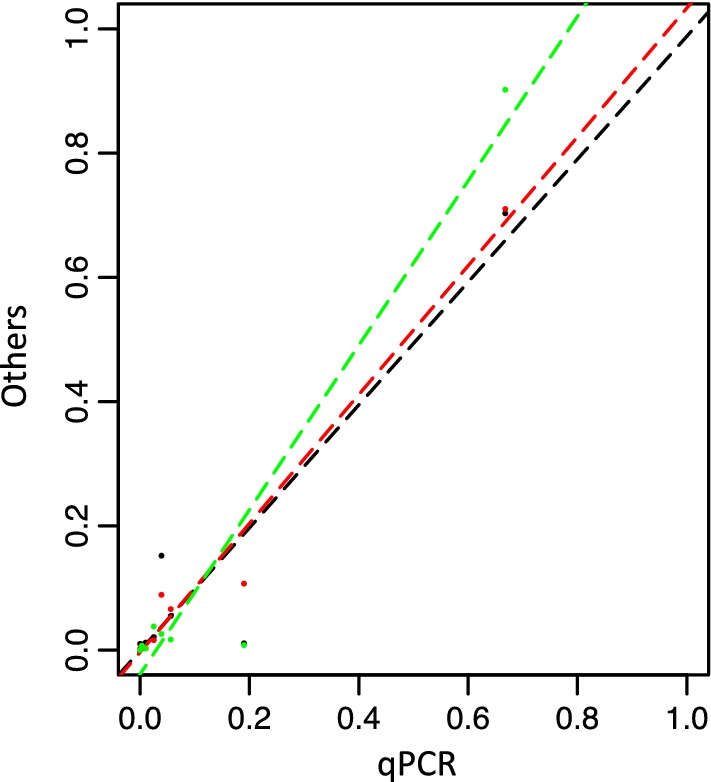
A quantitative comparison of the methods, with reference to quantitative PCR. The amplicon sequencing experiments (EM primers in red, NAR primers in green) yield similar results to that of the gene capture (in black). Values correspond to the relative abundance of the 8 bacterial symbionts detected in the pea aphid sample. Dashed lines represent linear regressions

We then investigated whether the two approaches produced different quantitative pictures. To this end, we counted the number of reads attributed to the different taxa and compared those numbers to independent evaluations based on quantitative PCR (Additional file 3: Table S2 and Fig. 2). With all the methods, the most abundant OTU belongs to *B. aphidicola*, the primary endosymbiont of the pea aphid. More generally, the different methods provided very similar results (Fig. 2), that were strongly correlated with the qPCR data (Pearson’s product-moment correlation, p < 0.001 for all the methods). We however noted large discrepancies in the abundance of *Spiroplasma*, that could result from different affinities of the primers/probes used in the various experiments to this bacterial taxon (class of *Mollicutes*). We conclude that on this particular genomic extract, although we did not a priori focus on specific bacterial clades, both amplicon sequencing and targeted gene capture produce comparable and reliable quantitative pictures.

Two conclusions can be drawn from this study. First, the amplicon sequencing approach, while potentially biased by universal primer pair affinity, can yield both qualitatively and quantitatively reliable information about microbial composition in a low complexity sample. Second, the gene capture approach, while much less used at the moment, produces reliable results, with potentially better performance for taxonomic assignment.

## Limitations

This study indicated similar performances of amplicon sequencing and gene capture to describe quantitatively and qualitatively the microbial diversity of an insect sample. However, we stress that this conclusion might not hold in samples comprising a larger diversity of bacterial taxa, especially if some taxa are more or less well amplified by a given set of PCR primers. In those conditions, gene capture might be more robust [6]. In addition, one should keep in mind that the analysis was intentionally limited to the region of the 16S gene covered by the amplicon sequencing approach. The region assembled through the gene capture approach is generally larger and can provide finer scale taxonomic assignment and phylogenetic resolution [6].

## Additional files

**Additional file 1: Table S1.** Primers used for quantitative PCR.

**Additional file 2.** Details on sequence data processing.

**Additional file 3: Table S2.** Normalized and relative abundances of the different OTUs.

## Abbreviation

EM: earth microbiome primer pair
