# Supplementary material for: Comparing 16S rDNA amplicon sequencing and hybridization capture for pea aphid microbiota diversity analysis
Source: BMC Res Notes. 2018 Jul 11;11:461. doi: 10.1186/s13104-018-3559-3 (PMC6042230; doi:10.1186/s13104-018-3559-3)
Supplement: Supplementary file 1 — Additional file 1: Table S1. Primers used for quantitative PCR. [file 13104_2018_3559_MOESM1_ESM.docx]

**Table S1.** Primers used for quantitative PCR

| Symbionts/aphid host | Primers names | Primers sequences (5’-3’) |
| --- | --- | --- |
| *Regiella insecticola* | Reg-EFTU-F | CGTGGAACCAAACGTGAAGAG |
|  | Reg-EFTU-R | TTTCAACCCCTTCTGGCAAC |
| *Hamiltonella defensa* | gyrBT-type484F | TTCCTGAAATCCATCGTTCC |
|  | gyrBT-type685R | CAAACGCAACGATCAAGAAA |
| *Rickettsiella viridis* | P136Ric-211F | GGGCCTTGCGCTCTAGGT |
|  | P136Ric-470R | TGGGTACCGTCACAGTAATCGA |
| *Rickettsia sp.* | RicCS-AF | TTTAGGCTAATGGGTTTTGGTCAT |
|  | RicCS-AR | TGCCCAAGTTCTTTTAACACCTC |
| *Spiroplasma sp.* | SPI618F | GTGGCAAGCGTTATCCGGAT |
|  | SPI834R | CCCACGCTTTCGTGCCACAA |
| *Buchnera aphidicola* | BuchDNAK-F | ATGGGTAAAATTATTGGTATTG |
|  | BuchDNAK-R | ATAGCTTGACGTTTAGCAGG |
| *Serratia symbiotica* | PASSGroE-AF1 | CCTCAAGGCTGTGGCCG |
|  | PASSGroE-AR1 | GAGTTTGCAGAGATGGTGCCTA |
| *Fukatsuia* (PAXS or X-type) | *PAX650F* | AAGGGCACGTAGGCGGTTTC |
|  | *PAX824R* | CGCACCTCAGCGTCAGTCTC |
| *Acyrthosiphon pisum* (aphid host) | Ap-CtpL-F | TACGAATCCGGCGAAGAAAC |
|  | Ap-CtpL-R | ATCACCACCAGCCAAACTGG |
