# Supplementary material for: Comparing 16S rDNA amplicon sequencing and hybridization capture for pea aphid microbiota diversity analysis
Source: BMC Res Notes. 2018 Jul 11;11:461. doi: 10.1186/s13104-018-3559-3 (PMC6042230; doi:10.1186/s13104-018-3559-3)
Supplement: Supplementary file 2 — Additional file 2. Details on sequence data processing. [file 13104_2018_3559_MOESM2_ESM.docx]

### Sequence data processing

Quality control and filtering of the raw reads were performed using the PRINSEQ-lite PERL script [1] as described in Denonfoux et al. [2]. An additional de-replication step was performed through PRINSEQ-lite for the SHS capture reads. The proportions of 16S rRNA gene sequences were estimated using SortMeRNA [3] with the default parameters (Table 1). Filtered data were then analysed using the Mothur software package v1.36 [4]. First, reads from the different approaches were aligned to the released version 119 of the SILVA small subunit rRNA sequence database [5]. The alignment was trimmed to the shared V4 hypervariable region defined from positions 537 to 686 on the *Escherichia coli* 16S rRNA gene. This step allowed selecting reads aligning on at least 50 bp to the target region. This first subset was further analysed to compare the three different sequence datasets (two amplicon datasets and one gene capture dataset). In brief, filtered sequences were pre-clustered using a threshold of 2 differences in order to remove sequences that likely contained pyrosequencing errors. Reads were screened for chimeras using UCHIME [6] and individually assigned to SILVA taxonomy (release 119) using RDP classifier [7]. Unassigned sequences and sequences belonging to non-prokaryotic domains, including chloroplasts, mitochondria and Eukaryotes, were removed. In order to avoid variation in power between the methods, random subsampling of sequences from each individual dataset was completed, to normalize the sequence count between datasets. Operational taxonomic units (OTUs) were created using a 3.0 % dissimilarity threshold and a nearest neighbour algorithm ignoring gaps. OTUs accounting for less than 1% of the total number of reads per sample were discarded. Finally, consensus taxonomic assignments of the remaining OTUs were obtained based on the SILVA database. To assess the reliability of each dataset, the same pipeline was applied to the different amplicon datasets individually with no restriction to the hypervariable V4 region.

# References:

1. Schmieder R, Edwards R. Quality control and preprocessing of metagenomic datasets. Bioinformatics. 2011;27:863 – 4.

2. Denonfoux J, Parisot P, Dugat-Bony E, Biderre-Petit C, Boucher D, Morgavi D, et al. Gene Capture Coupled to High-Throughput Sequencing as a Strategy for Targeted Metagenome Exploration. DNA Res. 2013;185–196.

3. Kopylova E, Noé L, Touzet H. SortMeRNA: Fast and accurate filtering of ribosomal RNAs in metatranscriptomic data. Bioinformatics. 2012;28:3211–7.

4. Schloss PD, Westcott SL, Ryabin T, Hall JR, Hartmann M, Hollister EB, et al. Introducing mothur: Open-source, platform-independent, community-supported software for describing and comparing microbial communities. Appl. Environ. Microbiol. 2009;75:7537–41.

5. Quast C, Pruesse E, Yilmaz P, Gerken J, Schweer T, Yarza P, et al. The SILVA ribosomal RNA gene database project: Improved data processing and web-based tools. Nucleic Acids Res. 2013;41:590–6.

6. Edgar RC, Haas BJ, Clemente JC, Quince C, Knight R. UCHIME improves sensitivity and speed of chimera detection. Bioinformatics. 2011;27:2194–200.

7. Wang Q, Garrity GM, Tiedje JM, Cole JR. Naïve Bayesian classifier for rapid assignment of rRNA sequences into the new bacterial taxonomy. Appl. Environ. Microbiol. 2007;73:5261–7.
