# Supplementary material for: Comparing 16S rDNA amplicon sequencing and hybridization capture for pea aphid microbiota diversity analysis
Source: BMC Res Notes. 2018 Jul 11;11:461. doi: 10.1186/s13104-018-3559-3 (PMC6042230; doi:10.1186/s13104-018-3559-3)
Supplement: Supplementary file 3 — Additional file 3: Table S2. Normalized and relative abundances of the different OTUs. [file 13104_2018_3559_MOESM3_ESM.docx]

**Table S2**: Normalized and relative abundances of the different OTUs

| **OTU** | **EM primers** | **NAR primers** | **Gene capture** | **qPCR** |
| --- | --- | --- | --- | --- |
| *Buchnera* | 1566 (71.0%) | 1994 (90.2%) | 1522 (72.3%) | 86.2 (66.8%) |
| *Regiella* | 16 (0.7%) | 15 (0.7%) | 20 (0.9%) | 0.4 (0.3%) |
| *Hamiltonella* | 197 (8.9%) | 58 (2.6%) | 328 (15.6%) | 5 (3.9%) |
| *Fukatsuia* | 6 (0.3%) | 6 (0.3%) | 25 (1.2%) | 1.9 (1.5%) |
| *Rickettsia* | 35 (1.6%) | 83 (3.8%) | 46 (2.2%) | 3.3 (2.6%) |
| *Rickettsiella* | 145 (6.6%) | 37 (1.7%) | 120 (5.7%) | 7.3 (5.7%) |
| *Serratia* | 5 (0.2%) | 1 (0.05%) | 22 (1.0%) | 0.1 (0.1%) |
| *Spiroplasma* | 236 (10.7%) | 17 (0.8%) | 23 (1.1%) | 24.9 (19.3%) |

Normalized abundances correspond to number of reads (for amplicon sequencing and capture) and to the ∆∆Cp for quantitative PCR. Relative abundances are given in parenthesis.
